# QSOX1: A Mysterious Golgi-Localized Disulfide Bond Catalyst and an Emerging Cancer Regulator

**DOI:** 10.3390/cancers18020339

**Published:** 2026-01-21

**Authors:** Shike Wang, Guan-Yu Xiao, Xiaochao Tan

**Affiliations:** 1 Department of Integrative Biology and Pharmacology, McGovern Medical School, The University of Texas Health Science Center at Houston, Houston, TX 77030, USA; shike.wang@uth.tmc.edu; 2 Department of Toxicology and Cancer Biology, The University of Kentucky, Lexington, KY 40536, USA; guan-yu.xiao@uky.edu

**Keywords:** QSOX1, cancer, deregulation, biomarker, therapeutic target

## Abstract

Quiescin sulfhydryl oxidase 1 (QSOX1) is a disulfide bond-forming enzyme with both disulfide isomerase and oxidoreductase activities. It plays an important role in protein folding, stability, and secretion. Growing evidence demonstrates that QSOX1 is upregulated in multiple cancer types and influences key behaviors of cancer cells, including proliferation, migration, invasion, and metastasis. Elevated QSOX1 expression is also associated with tumor malignancy and disease relapse. However, the molecular mechanisms by which QSOX1 drives cancer progression remain incompletely understood. In this review, we summarize current knowledge of QSOX1 expression and regulation in cancer, discuss its functional roles, and highlight key unanswered questions to warrant further investigation.

## Simple Summary

In this Commentary, we summarize recent advances in understanding the role of the Golgi-localized disulfide bond catalyst QSOX1 in human cancers. Disulfide bond formation is critical for proper protein folding and stability, and its dysregulation contributes to cancer progression by modulating extracellular matrix (ECM) organization and cell signaling. QSOX1 is a unique oxidoreductase that catalyzes de novo disulfide bond formation primarily in the Golgi apparatus and extracellular space, distinguishing it from classical ER-resident enzymes. QSOX1 is upregulated in many cancers through a combination of genetic, transcriptional, microenvironmental, and post-transcriptional mechanisms. Functionally, QSOX1 can promote tumor progression by enhancing matrix metalloproteinase activity, supporting cancer cell survival under oxidative stress, regulating ECM assembly, and fostering immune-suppressive tumor microenvironments. However, QSOX1 also exhibits tumor-suppressive functions in certain cancer contexts, underscoring its context-dependent roles. Although QSOX1 is emerging as a potential biomarker and therapeutic target, further studies are needed to define its regulatory mechanisms, substrates, and isoform-specific functions to enable effective clinical translation.

## 1. Introduction

Disulfide bond formation is one of the most common post-translational modifications in proteins and plays a critical role in ensuring proper protein folding, stability, and function in both intracellular and secreted proteins [1]. In recent years, growing evidence in cancer models has highlighted that dysregulated disulfide bond formation not only alters the stability and secretion of pro-tumorigenic proteins but also shapes the mechanical properties of the extracellular matrix (ECM), modulates receptor signaling, and supports metastatic competence [2,3,4,5].

Disulfide bond formation occurs primarily in the endoplasmic reticulum (ER) during oxidative protein folding and is mediated by protein disulfide isomerases (PDIs) [6]. Disulfide bonds in the ER are introduced co- and post-translationally by protein PDIs through a redox reaction in which the disulfide bond within PDI’s CXXC active site is reduced as it oxidizes substrate proteins. To sustain this catalytic cycle, ER Oxidoreductin-1α/β (ERO1α/β) re-oxidizes reduced PDI by accepting electrons and transferring them to molecular oxygen, producing H_2_O_2_ as a by-product. This replenishes oxidized PDI, enabling continuous rounds of disulfide bond formation [7].

**Figure 1:**
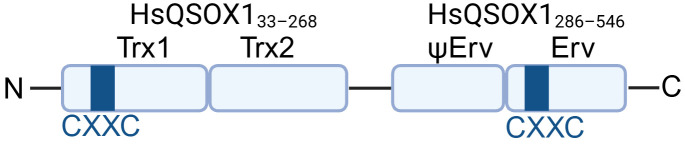
A schematic illustration of QSOX1 domains. QSOX1 is composed of thioredoxin-like (Trx) domains and sulfhydryl oxidase (Erv) domains connected by a linker. Trx1 contains a redox-active CXXC motif responsible for substrate oxidation and disulfide bond formation. ΨErv represents a degenerated sulfhydryl oxidase domain [13], whereas the Erv domain contains a redox-active CXXC motif essential for de novo disulfide bond formation. C indicates cysteine. XX represents any amino acids other than cysteine.

Quiescin Sulfhydryl Oxidases (QSOXs) are a small family of flavin adenine dinucleotide (FAD)-dependent oxidoreductases. They are conserved across metazoans and share a characteristic domain organization, including an N-terminal thioredoxin-like domain and a C-terminal FAD-dependent oxidase domain [8] (Figure 1), which together enable efficient electron transfer for oxidative protein folding. To date, QSOX1 is the only confirmed Golgi-resident enzyme that can directly catalyze de novo disulfide bond formation [9], while its homology QSOX2 is much less studied. Unlike PDIs that rely on ERO1 for reoxidation in the ER, QSOX1 integrates both substrate oxidation and electron transfer within a single enzyme [10]. Furthermore, unlike other ER-resident oxidoreductases, QSOX1 is predominantly active outside the ER, carrying out most of its catalytic function in the Golgi apparatus and the extracellular space, allowing it to catalyze de novo disulfide bond formation after proteins leave the ER [9,11,12]. This distinctive functionality positions QSOX1 as a key regulator of extracellular matrix assembly, protein stability, and tumor-stromal interactions in cancer.

Emerging evidence indicates that QSOX1 exhibits context-dependent expression and function across different cancer types [14,15,16,17,18,19]. This review aims to provide an overview of current mechanistic insights into QSOX1 and highlight its significance as a potential biomarker and therapeutic target in cancer.

## 2. Mechanisms Regulating Aberrant QSOX1 Expression in Cancer

Transcriptomic and proteomic analyses have consistently shown that QSOX1 is upregulated at both the mRNA and protein levels in multiple cancers, including pancreatic ductal adenocarcinoma [20], breast carcinoma [21], prostate cancer [22], lung cancer [23], and glioblastoma [24]. *QSOX1* is located on chromosome 1q25.2, a region that is frequently amplified in several cancer types [25,26]. Genomic analyses reveal that copy-number alterations and focal amplifications of the *QSOX1* locus vary widely across cancer types-from rare amplification in renal cell carcinoma (~1.1%) to frequent amplification in breast cancer (~37.4%) [27]. This variability suggests that locus amplification alone cannot fully account for the elevated QSOX1 expression observed in many tumors, underscoring the importance of non-genetic regulatory mechanisms.

Consistent with a role for non-genetic regulation, multiple signaling pathways implicated in tumor progression have been shown to transcriptionally regulate QSOX1 expression. Previous studies have shown that *QSOX1* transcription is activated in cancers. NK3 Homeobox 1 (NKX3.1) functions primarily as a tumor suppressor, particularly in prostate cancer, where its loss or reduced expression disrupts epithelial differentiation, increases genomic instability, and drives proliferation and tumor progression [28]. Two studies from the Abdulkadir group further demonstrated that *QSOX1* is a direct transcriptional target of NKX3.1 in prostate cancer, and that loss of NKX3.1 leads to elevated QSOX1 expression [29,30]. In addition, oncogenic signaling pathways also regulate *QSOX1* transcription. Identification of a Signal Transducer and Activator of Transcription (STAT)-binding site within the *QSOX1* locus suggests that QSOX1 may be under direct transcriptional control of the STAT signaling pathway [31]. Transforming Growth Factor Beta (TGF-β), a master regulator of ECM remodeling and epithelial-to-mesenchymal transition (EMT), also upregulates QSOX1, particularly in fibroblasts and mesenchymal-like cancer cells, thereby reinforcing tumor-stroma interactions [32,33]. Two hypoxia-response elements (HREs) have been identified in the *QSOX1* promoter, and transcription factor hypoxia-inducible factor-1 (HIF-1) can directly bind these sites [34]. Experimental evidence in pancreatic cancer cells shows that QSOX1 expression depends on HIF-1 under hypoxic conditions [34]. However, a preprint study by the Gleghorn group did not reproduce this hypoxia-dependent induction of QSOX1, despite successfully inducing HIF-1 in the same cell line. Instead, they found that QSOX1 expression was strongly upregulated by culture on stiff surfaces such as plastic or glass, but not on soft polyacrylamide gels [35]. In addition, increased QSOX1 expression has been observed in response to oxidative stress induced by pericellular hydrogen peroxide (H_2_O_2_) or iron in both PC12 and MCF-7 cells [36]. These observations indicate that QSOX1 expression is responsive not only to intrinsic oncogenic signaling but also to extracellular and mechanical stimuli.

Post-transcriptional mechanisms also contribute to the regulation of QSOX1. RNA 5-methylcytosine (m5C) modification plays a critical role in the pathogenesis of many tumor types [37]. RNA-seq and m5C-BisSeq analyses conducted by the Tian lab identified QSOX1 as a downstream target of aberrant m5C modification. Specifically, the m5C writer NOP2/Sun RNA methyltransferase family member 2 (NSUN2) deposits m5C within the QSOX1 coding sequence, which in turn enhances QSOX1 translation through recognition by the m5C reader Y-box binding protein 1 (YBX1) in gefitinib-resistant EGFR-mutant non-small cell lung cancer cells [38].

**Figure 2:**
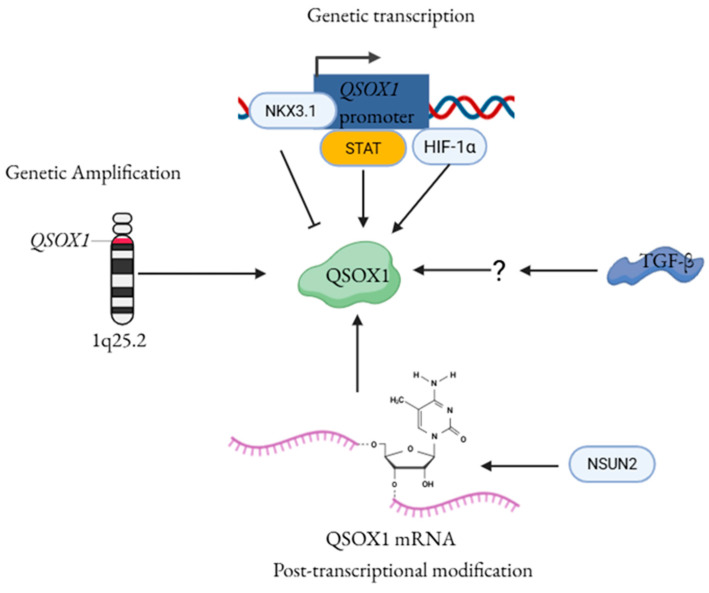
An illustration summarizing the current understanding of QSOX1 regulatory mechanisms at the genetic, post-transcriptional levels. ↑ indicates promotion, and ┴ indicates inhibition. ? indicates unknown mechanism. Abbreviations: QSOX1—Quiescin sulfhydryl oxidase 1; NKX3.1—NK3 Homeobox 1; STAT—Signal Transducer and Activator of Transcription; HIF-1α—Hypoxia-Inducible Factor 1-alpha; TGF-β—Transforming Growth Factor Beta; NSUN2—NOP2/Sun RNA methyltransferase family member 2.

Collectively, these findings suggest that QSOX1 expression is regulated through a complex network that extends beyond genetic alterations to encompass transcriptional, microenvironmental, and post-transcriptional mechanisms (Figure 2). This coordinated regulation suggests that QSOX1 expression is dynamically adapted to the evolving demands of the tumor microenvironment. Importantly, such regulatory plasticity positions QSOX1 not merely as a passive marker of malignancy, but as an actively controlled effector whose expression is tightly coupled to the function of cancer cells.

## 3. The Biological Function of QSOX1 in the Context of Cancer

QSOX1’s impact on cancer biology is highly context-specific, with evidence supporting both pro-tumorigenic and tumor-suppressive functions, depending on the cancer type and potent subsequent signaling pathways (Table 1). Because QSOX1 localizes to both the Golgi apparatus and the extracellular space, its complex function arises from a combination of intracellular functions and extracellular activities. The mechanisms through which QSOX1 mediates cancer progression can be broadly grouped into several major functional categories.

### 3.1. QSOX1 Mediates Activation of Matrix Metalloproteinases (MMPs)

QSOX1 is highly expressed in pancreatic ductal adenocarcinoma [20] (PDAC) and luminal-like breast cancer cell lines [15], particularly elevated levels of the short isoform. In both cancer types, QSOX1 promotes cell proliferation, migration, and invasion. A key conserved finding across studies is that QSOX1 enhances the enzymatic activity of MMP2 and MMP9 without altering their transcription, indicating a post-transcriptional or post-secretory mode of regulation. However, a major unresolved question is how QSOX1 modulates MMP activity at the molecular level. It remains unclear whether MMPs are direct substrates of QSOX1-mediated disulfide bond formation or whether QSOX1 influences MMP function indirectly through other modulators of MMPs. In addition, the specific compartment in which QSOX1 influences MMP activity (e.g., the Golgi lumen, extracellular space, or pericellular space) also remains to be determined.

### 3.2. QSOX1 Enhances Tumor Cell Survival Under Oxidative Stress

Oxidative stimuli upregulate QSOX1 expression, and in turn, elevated QSOX1 can protect cancer cells from oxidative-stress-induced apoptosis [36]. This protective effect reflects QSOX1’s ability to support redox balance during protein folding and may contribute to the survival of cancer cells under conditions such as hypoxia, inflammation, or metabolic stress. Thus, QSOX1 can function as part of an adaptive stress-response mechanism that enhances tumor resilience.

### 3.3. QSOX1 Modulates Laminin Maturation and Deposition

QSOX1 plays a critical role in ECM organization by regulating the incorporation and assembly of specific matrix components. Loss of QSOX1 selectively disrupts laminin integration into the ECM generated by quiescent fibroblasts, while exerting limited effects on collagen IV or fibronectin assembly [9]. Mechanistic work further shows that QSOX1 facilitates the formation of laminin heterotrimers, highlighting a direct enzymatic role in matrix assembly.

A recent study demonstrates that QSOX1 is enriched on laminin-coated surfaces, but only minimally detected on collagen- or gelatin-coated surfaces, supporting the specificity of its activity [42]. This study also shows that QSOX1 binds to cysteine-rich fibronectin, although whether fibronectin is a QSOX1 substrate remains elusive. Collectively, these findings suggest that QSOX1 plays a role in ECM remodeling by facilitating the proper assembly and incorporation of specific ECM proteins, thereby shaping a microenvironment that supports invasion and metastasis.

### 3.4. QSOX1 Influences Paracrine Signaling and Immune Modulation

Within the tumor microenvironment, cancer-associated fibroblasts secrete QSOX1, resulting in the localized production of H_2_O_2_. This redox signal activates STAT3 signaling in adjacent dormant cancer stem cells, resulting in upregulation of the immune checkpoint molecule PD-L1 [31]. Elevated PD-L1 expression suppresses CD8^+^ T-cell infiltration and cytotoxic activity, thereby promoting immune evasion. Through this STAT3-dependent paracrine redox signaling axis, stromal-derived QSOX1 establishes an immune-suppressive niche that supports cancer cell dormancy and long-term tumor persistence.

### 3.5. QSOX1 Functions as a Tumor Suppressor

Although most studies support a pro-tumorigenic role for QSOX1, several reports describe context-dependent tumor-suppressive functions. In invasive ductal carcinoma of the breast, high QSOX1 expression has been inversely associated with tumor aggressiveness. QSOX1 overexpression reduces MMP-2 activity and significantly suppresses tumor growth in vivo [14]. In hepatocellular carcinoma (HCC), QSOX1 can enhance susceptibility to oxidative stress by reducing NRF2 activation, thereby lowering the cellular antioxidant capacity. As a result, QSOX1 sensitizes HCC cells to oxidative injury, exhibiting a tumor-suppressive role in this specific context [40,43].

## 4. Diagnostic and Therapeutic Potential of QSOX1 in Cancer

### 4.1. QSOX1 as a Biomarker in Cancer Diagnosis

Liquid biopsy has become an increasingly useful tool in cancer diagnostics. Multiple studies have demonstrated that QSOX1 can be secreted by either cancer cells or stromal fibroblasts. Clinically, elevated QSOX1 peptides have been detected in the blood of patients with several malignancies, including lung cancer [45], pancreatic Cancer [46] and HCC [43]. Notably, in early-stage HCC, higher serum levels of core-fucosylated QSOX1 (cf-QSOX1), as well as increased tumor QSOX1 expression, correlate with a longer time to recurrence and improved overall survival, suggesting a favorable prognostic value in this specific context. However, the clinical significance of circulating QSOX1 levels in other cancer types, including lung cancer, remains unclear. Given the limited studies to date, it remains unclear whether QSOX1 can serve as a general biomarker across multiple cancer types or whether its utility is restricted to specific disease contexts.

### 4.2. QSOX1 as a Therapeutic Target for Cancer Treatment

For cancer types in which QSOX1 exhibits pro-tumorigenic activity, disruption of QSOX1 markedly suppresses cancer cell proliferation, migration, and invasion in vitro, and significantly reduces tumor growth and metastasis in vivo. Consistently, pharmacologic inhibition of QSOX1 using small-molecule inhibitors- SBI-183 [47] and Ebselen [48]— or a monoclonal antibody (MAb492.1) results in comparable suppression of tumor progression [33,49]. These findings collectively highlight QSOX1 as a promising therapeutic target for cancer treatment, while also warranting the need to consider context-dependent effects of QSOX1 inhibition.

## 5. Discussion

Despite growing evidence linking QSOX1 to cancer progression, the mechanisms that regulate its expression and activity remain largely unclear. For instance, 3′-RACE PCR has identified a *QSOX1* mRNA variant containing an extended 3′-UTR, although no corresponding alternative protein isoform has been detected [21]. This finding suggests that post-transcriptional regulation through the 3′-UTR, potentially involving RNA-binding proteins or microRNAs, may modulate QSOX1 stability or translation; however, these regulatory mechanisms have yet to be thoroughly investigated. QSOX1 exists as a long (QSOX1-L, 747 amino acids) and a short (QSOX1-S, 604 amino acids) isoform generated by alternative splicing of exon 12. The two isoforms differ primarily at their C-termini, with the long isoform containing a transmembrane domain that is absent in the short isoform [50]. Given that QSOX1-L can undergo proteolytic cleavage and be secreted into the extracellular space [9], similar to QSOX1-S, it is likely that both isoforms exert comparable functions in the extracellular environment. However, the presence of a transmembrane anchor on QSOX1-L but not on QSOX1-S suggests that the isoforms may differ in their intracellular localization, regulation, and function, aspects that remain poorly explored. In addition, the splicing factors that regulate QSOX1 alternative splicing are currently unknown.

**Figure 3:**
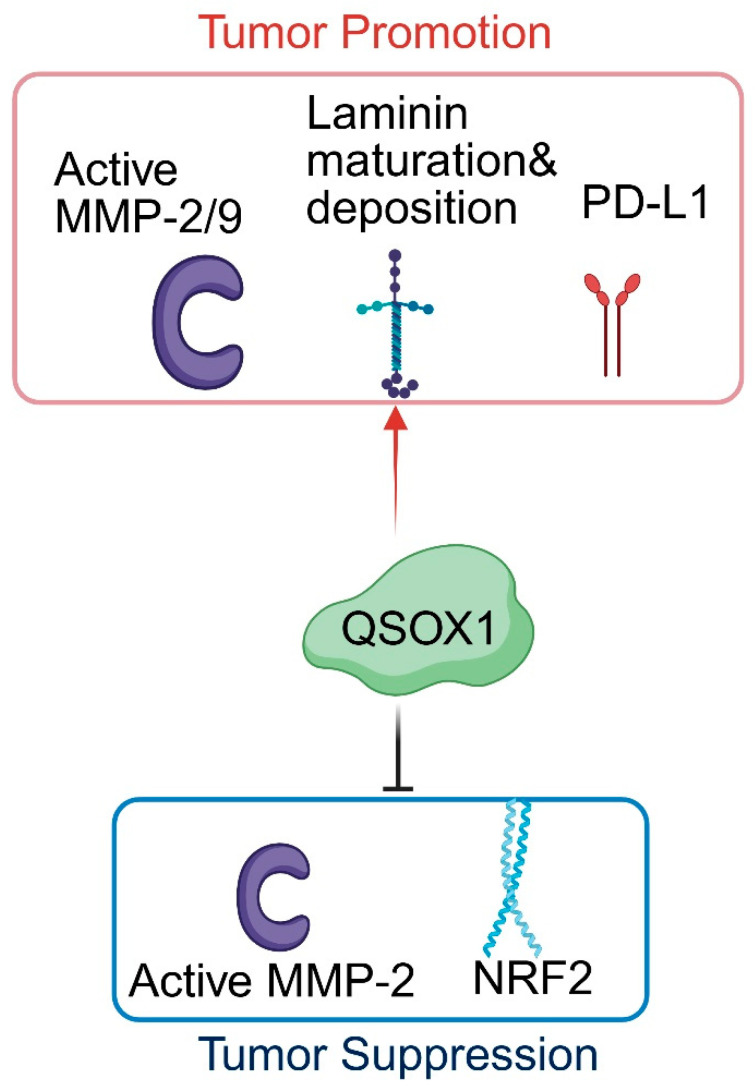
A schematic depicting QSOX1’s role in cancer progression. In its tumor-promoting role, QSOX1 enhances MMP-2/9 activity, promotes laminin maturation and deposition in the ECM, and upregulates PD-L1 expression. In its tumor-suppressive role, QSOX1 inhibits MMP-2 activity and NRF2 activation. ↑ indicates promotion, and ┴ indicates inhibition. List of abbreviations: MMP—Matrix Metalloproteinase; PD-L1—Programmed Death-Ligand 1; NRF-2—Nuclear Factor Erythroid 2-Related Factor 2.

Extensive mechanistic work from Dr. Fass’s group over the past two decades has provided key insights into QSOX1 biology. Their studies demonstrate that QSOX1 regulates extracellular matrix composition by promoting the proper assembly and deposition of laminin, establishing a direct role for QSOX1 in ECM organization [9]. They also identified ZG16 as a potent substrate of QSOX1 in colon cancer [51]; however, the biological relevance of ZG16 in cancer remains unclear. Other groups have shown that QSOX1 can modulate the activity of MMP2 and MMP9, yet these enzymes likely represent only a small fraction of the substrates affected by QSOX1’s disulfide-bond-forming activity. Overall, the molecular mechanisms by which QSOX1 promotes cell proliferation, motility, and ECM remodeling remain poorly defined, and the full spectrum of QSOX1 substrates-both intracellular and extracellular- has not been comprehensively characterized. Notably, while QSOX1 enhances invasion and metastasis in cancers such as PDAC, breast cancer, and lung cancer, it displays tumor-suppressive properties in HCC and in certain breast cancer contexts (Table 1 and Figure 3). Defining QSOX1 substrates and their functional consequences will be essential for understanding how this enzyme contributes to cancer biology, particularly given its context-dependent roles across different tumor types. More recently, Dr. Fass and colleagues uncovered a surprising intracellular role for QSOX1 in the formation of disulfide bonds within specific Golgi glycosyltransferases, including ST6GAL1, ST3GAL1, and B3GALT5 [52]. These enzymes are critical for core glycan synthesis and overall Golgi function [53,54]. However, the extent to which QSOX1-mediated regulation of glycosyltransferases affects oncogenic secretion, glycoprotein maturation, or vesicular transport in the Golgi remains unknown and warrants further investigation.

## 6. Conclusions

In conclusion, although QSOX1 has emerged as a critical regulator of protein folding, extracellular matrix organization, and tumor–microenvironment interactions, its molecular mechanisms of action remain incompletely understood. Elucidating how QSOX1 expression and activity are regulated, identifying its full repertoire of intracellular and extracellular substrates, and defining the functional differences between its isoforms will be crucial for clarifying its context-dependent roles in cancer. Such knowledge will not only deepen our understanding of Golgi-centered redox biology but also provide a rational framework for the development of QSOX1-based biomarkers and targeted therapeutic strategies tailored to specific cancer types.

## Figures and Tables

**Table 1:** A table summarizing the currently identified functions of QSOX1 in cancer.

Cancer Types	QSOX1 Expression	Effect on Cancer Cells	Potential Mechanisms	References
Breast Cancer	↑	Reduce cell proliferation and invasion, increase cell adhesion	MMP-2 activity ↓, p62 ↑, Autophagosome/Lysosome Fusion ↓	[14] [39]
	↑	Cell proliferation and invasion	MMP-9 activity ↑	[15]
Lung Cancer	↑	Gefitinib resistance	Unknown	[38]
	↑	Cell proliferation, migration, invasion	Unknown	[40]
Esophageal cancer	↑	Cancer stem cell dormancy	ROS ↑, PD-L1 ↑, CD8^+^ T cells infiltration ↓	[31]
Glioblastoma	↑	Cell proliferation, colony formation, invasion	PI3K/Akt pathway ↓	[41]
HCC	↑	Reduce cell invasion and metastasis	Integrinβ1/FAK and EGFR/Raf/ERK signaling pathway ↓	[42]
		Ferroptosis	ROS ↑, EGFR activation ↓, NRF2 activation ↓	[43]
		Anoikis	Intracellular fatty acids and cholesterol ↓	[44]
PDAC	↑	Cell proliferation and invasion	MMP-2/9 activity ↑	[20]

↑ indicates upregulation; ↓ indicates downregulation.

## Data Availability

No new data were created or analyzed in this study. Data sharing is not applicable to this article.
